# “Are you accepting new patients?” A pilot field experiment on telephone‐based gatekeeping and Black patients’ access to pediatric care

**DOI:** 10.1111/1475-6773.13089

**Published:** 2018-12-03

**Authors:** Tamara G.J. Leech, Amy Irby‐Shasanmi, Anne L. Mitchell

**Affiliations:** ^1^ Montclair State University Montclair New Jersey; ^2^ University of West Georgia Carrollton Georgia; ^3^ Office of Equal Opportunity IUPUI Indianapolis Indiana

**Keywords:** sociology, access/demand/utilization of services, child and adolescent health, determinants of health/population health/socioeconomic causes of health, racial/ethnic differences in health and health care

## Abstract

**Study Objectives:**

To determine whether name and accent cues that the caller is Black shape physician offices’ responses to telephone‐based requests for well‐child visits.

**Method and Data:**

In this pilot study, we employed a quasi‐experimental audit design and examined a stratified national sample of pediatric and family practice offices. Our final data include information from 205 audits (410 completed phone calls). Qualitative data were blind‐coded into binary variables. Our case‐control comparisons using McNemar's tests focused on acceptance of patients, withholding information, shaping conversations, and misattributions.

**Findings:**

Compared to the control group, “Black” auditors were less likely to be told an office was accepting new patients and were more likely to experience both withholding behaviors and misattributions about public insurance. The strength of associations varied according to whether the cue was based on name or accent. Additionally, the likelihood and ways office personnel communicated that they were not accepting patients varied by region.

**Conclusions:**

Linguistic profiling over the telephone is an aspect of structural racism that should be further studied and perhaps integrated into efforts to promote equitable access to care. Future research should look reactions to both name and accent, taking practice characteristics and regional differences into consideration.

## 1 INTRODUCTION

Well‐child visits are considered the “foundation of preventive pediatrics.”^1^ Consistent access to these types of visits can contribute to a reduction in long‐term health issues^2^ in addition to preventing or reducing emergency care and hospitalizations.^3^, ^4^ However, there are substantial racial disparities in the proportion of children who regularly receive preventive care in general and well‐child visits in particular.^5^, ^6^

Differential access to well‐child visits is, therefore, an especially relevant subcategory of differential access to health care services. The Health Care Access Barriers Model emphasizes three categories of access barriers that contribute to racial inequities in access to health care services: financial (eg, insurance coverage), structural (eg, geographic proximity and wait times), and cognitive barriers.^7^ The present study focuses on cognitive barriers. Extant literature mainly frames cognitive barriers as either patient knowledge of disease prevention/care or patient‐provider communication.^7^ A large body of research indicates that patient‐provider communication serves as a barrier for racial minorities, calling for training physicians and nurses in communication skills.^8^, ^9^, ^10^ However, scholars have paid scant attention to the likely first interaction between a prospective patient and provider offices: the initial telephone inquiry.

Focusing on the initial telephone inquiry highlights that, regarding *accessing* care, scheduling staff may be the most important members of the provider office, as they serve as gatekeepers to services. These individuals could explicitly deny potential patients access to the office by indicating that the office is not accepting new patients. Additionally, if we extrapolate the information on physician‐patient communication to scheduler‐patient communication, we can expect staff's verbal behavior toward Black patients to be plagued with some of the problems currently documented in physician‐patient communication. However, because much of this interaction is over the phone, it would be dependent on verbal cues that the potential patient is Black.

These verbal cues do not occur in a social vacuum. Previous research finds that discrimination varies by demographic context (eg, concentration of the Black population and level of segregation) and by geographic region.^11^, ^12^, ^13^ For example, Cotton's^14^ research suggests that Blacks in the Western United States experience the greatest amount of discriminatory treatment in earnings, while educational gaps between Blacks and Whites are the largest in the Northeast. Pendergrass^15^ reports that regional differences in racial prejudice exist, but are nuanced. Her Black respondents believe that racial hostility is not confined to the South; instead, discrimination is simply more overt in the South when compared to the Northeast. However, they perceive the South as having more racially mixed environments than the Northeast and Midwest, as well as more economic opportunities. Overall, research suggests that racial discrimination may manifest itself in unique ways across the United States. So, while exploring the linguistic‐based relationship between office staff and potential pediatric patients sits at the core of our pilot study, we also pay special attention to geographic context.

### 1.1 Linguistic‐based racial discrimination in the health care setting

Accent‐ and name‐based discrimination is known to limit access to resources. Name and accent cues often facilitate discrimination and quick personal judgments in everyday interactions.^16^ The issue of linguistics has long been linked to discriminatory treatment, especially for those speaking Black‐accented English.^17^ Linguistic profiling—primarily through name and accent cues—can be considered the “auditory equivalent to visual racial profiling.”^18^ Existing literature shows that perceived White names typically receive higher more positive responses on job applications, including more callbacks, as compared to perceived Black and other ethnic names.^19^, ^20^, ^21^ Experimental audit studies (or field studies) have shown bias in over‐the‐phone interactions in housing, human resources, and insurance contexts based on the perceived race and class of the inquiring individual as determined by his/her accent and name.^19^, ^22^, ^23^

Existing research on the subject of name and linguistic‐based studies focuses mainly on profiling in the housing and job market, insurance agencies, and credit markets. There is a paucity of information on linguistic profiling and access to health care, but emerging evidence indicates that linguistic profiling may shape access to mental health care appointments.^24^ A recent phone‐based audit study investigated the accessibility of psychotherapist appointments based on responses to voicemails requesting appointments.^25^ Actors spoke in accented English and provided names to indicate that they were either Black or White. There were no racial differences among low‐income inquiries, but Black middle‐class help‐seekers were less likely to be offered an appointment with a therapist than White middle‐class help‐seekers.

However, provider offices’ influence over patients’ likelihood to schedule and attend appointments may not be limited to explicit statements regarding acceptance and rejection. In general, there is evidence that the race and ethnicity of patients influence providers’ communication inside the examining room by affecting information giving and withholding, dominating conversations, and misattributions (making assumptions/stereotypes about the patient).^26^, ^27^, ^28^ Scholars have long accepted that the nature of communication between provider and patient shapes adherence to medical advice,^29^, ^30^ including likelihood to attend follow‐up visits. These types of behaviors may be equally salient in staff‐patient interaction outside of the examining room, especially when patients are initially attempting to establish a relationship with (or access to) the office.

In the pilot study described here, we explore the shape and scope of differential access‐relevant communication between staff and patients based on linguistic cues suggesting that the patient is Black. Given that it is a pilot study, we have both aims and hypotheses. Our aims were to:

- Determine whether there is an association between aspects of the study design—differing scripts, ordering of calls, etc.—and outcomes of interest.
- Explore regional variations that might shape the way staff members enact gatekeeping behavior and therefore the way we should design samples and measure acceptance in future studies.
- Explore potential differences in the meaning of name cues vs linguistic cues (ie, Do they both merely serve as the same “cue to Blackness?” or Do they have different relationships to staff behavior?)

**Figure 1:**
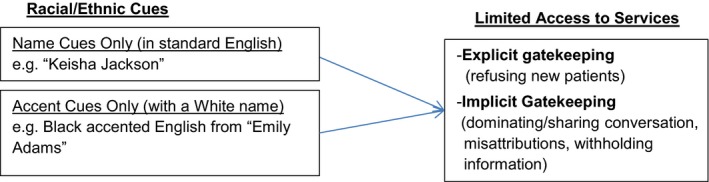
Conceptual model informing the study [Color figure can be viewed at http://www.wileyonlinelibrary.com/]

*Note:* Control is consistently “Emily Adams” in standard English

Based on existing literature, we also formed initial hypotheses that flow from the conceptual model presented in Figure 1. Compared to patients perceived to be White (the control patients), we hypothesize:

1. Parents of patients perceived to be Black will be told that offices are accepting patients less often.
2. Parents of patients perceived to be Black will be subjected to inquiries before their question is answered (ie, subjected to withholding information).
3. Parents of patients perceived to be Black will be asked questions about insurance coverage more often (ie, subjected to misattributions).
4. Patients perceived to be Black will be informed of potential restrictions/barriers to care more often (ie, staff dominating/shaping conversations).

## 2 METHODS

Between February and May of 2012, we implemented a national field experiment design using telephone audits. Field experiment approaches are widely used in the study of discrimination and have been valued as providing some of the most rigorous evidence of discriminatory behavior in areas ranging from employment^31^ to referrals to medical services.^32^ More specific to our study, telephone audits as a subset of these field experiments have been established as a useful methodology for the assessment of linguistic‐ and name‐based discrimination.^23^, ^33^ This type of approach maximizes scholars’ ability to isolate causal effects by carefully controlling comparisons.

### 2.1 Sample

The target population for the audits included offices that provide well‐child visits. Individual office staff members were not the study participants. Instead, we were interested in the provider office as an organism, in the aggregate, so we employed a stratified sampling method to randomly select provider office phone numbers included in the publicly available online Cigna database of national providers. This approach also allowed us to identify sample participants—that is, offices—by phone number without recording the actual office name.

Using this sampling base, we employed a stratified random sampling process. The sample for each of the experimental categories—name‐based and accent‐based—was drawn from provider offices in the three most and three least segregated metropolitan areas in each region (Northeast, South, Midwest, and West), according to the Black‐White segregation indices for the largest 100 metropolitan statistical areas (MSAs).^34^ Each phone number received five attempts before we removed it from the sample. Answering machines/voicemails received a disposition code, and each time an auditor reached one it was coded as an attempt, but no message was left.

Within each category in the sampling frame, we randomly selected from offices labeled as pediatric or family practice offices. We aimed to complete 25 audits, or 50 calls, within each of the eight sampling cells for a total of 200 audits. For logistical reasons (the need for all name‐based calls to be performed by White‐sounding auditors, and half of the accent‐based calls to be performed by White‐sounding callers), the design was for nine of the audits in each cell to be name‐based and 16 to be accent‐based. Because of some variation during the data collection process—including unanswered phones and the productivity of auditors—a total of 205 audits were completed, with a final breakdown as follows: 53 offices in the Northeast, 49 offices in the Midwest, 52 offices in the South, and 51 offices in the West. For our initial pilot, we did not record office characteristics such as size, length of time in practice. This type of data should be collected and taken into consideration in future studies.

### 2.2 Data collection

Before the team began collecting data, we validated the name and linguistic cues. First, we identified names for Black and White women based on names used in previous field audit studies.^19^, ^20^ Second, we gathered responses to our auditors’ recorded voices. All auditors were Black women, regardless of name and linguistic cues used in the experiment, but their natural speaking voices varied. After listening to a recording of an auditor's voice, email respondents were asked to describe the person speaking. We did not prompt respondents for the racial identity of the speaker. We hired auditors who were consistently described as African American or Black or who were consistently described as White by respondents who mentioned race. Thus, Black‐accented English is operationalized, in this study, as having a voice that others (of multiple races) perceive to be coming from a Black woman even when they cannot see the woman.

For the accent‐based audits, within 48 hours of each other, two auditors attempted to schedule an appointment with the same pediatric or family practice office. One auditor read a script in Black‐accented English and indicated that her name was, for example, Emily Adams. The other woman auditor read a similar script in standard English and stated that her name was, for instance, Sara Novak.

We used the same process for name‐based audits. In this case, one auditor indicated that her name was Keisha Jackson or Ebony Williams and the other stated that her name was Emily Adams or Sara Novak. We randomly varied the order of calls. We also used slightly different scripts, so as not to raise suspicion of two identical calls within 48 hours, and systematically varied between three scripts.

We conducted extensive training with auditors to ensure they provided standardized responses to common questions. For example, when a provider's office asked whether an auditor had insurance, the auditor would reply “yes,” but would not volunteer the type of insurance. If/when a provider's office asked what insurance an auditor had, she would reply “Cigna.”

A computer‐assisted telephone interviewing (CATI) system was used to dispense the sample and maintain administrative data. All auditors kept detailed handwritten notes on the telephone exchange that served as traditional field note jottings. Once the call ended, they immediately expanded these jottings into typed, nearly verbatim data on the conversation. The field notes only included words in the discussion—we did not include any indicators of tone or inflection, nor information about the auditor's feelings or interpretations. The average call lasted 46 seconds. The fact that the auditor did not record any subjective data and that the exchanges were extremely short decreased the chance that any auditor would bias the results. Also, the shift supervisor listened in to 1 in every two calls (via the “spy” phone) to provide overall quality checks of the data collected.

### 2.3 Measures

#### 2.3.1 Independent variables

There are two independent variables: *name‐cue* (coded 1 for Black and 0 for control) and **accent‐cue** (coded one as 1 for Black and 0 for control).

#### 2.3.2 Study design variables

We treat the extent of *segregation*—at the level of the metropolitan area—as a potentially moderating variable. We coded 1 for high‐segregation areas and 0 for low‐segregation areas. We also used three different scripts. *Script* is treated as a categorical variable, and throughout the analysis, comparisons are made to script one. *Order of call* is a dichotomous variable and refers to whether the call was the first (1) or second (0) call in the audit. The team maintained all of these data within the CATI system. Finally, *region is a categorical variable that* indicates whether the office was located in the West, Northeast, Midwest, or South.

#### 2.3.3 Dependent variables

*Acceptance* of patients is a dichotomous variable representing responses to “Are you accepting new patients?” A response of “Yes” is coded as 1; other responses are coded as “0.” This variable serves as our indicator of explicit gatekeeping or discrimination. For exploratory purposes, we looked further into all of the answers coded as “0” in this variable. We separated all of these answers that did not receive an immediate response of “yes” into three categories that we term *reluctant acceptance* (a response of “yes, but…”), *implicit rejection* (never giving an answer of yes or no) and *explicit rejection* (a response of “no”).

We also focused on other indicators of gatekeeping activity. If the person answering the phone posed his/her own question before answering the auditor's inquiry of “Are you accepting new patients?”, it was an indicator of *withholding information (coded dichotomously)*. If the office representative emphasized any conditions or qualifications for acceptance (eg, “Well, we can only see you if you can get us his records from the previous doctor”) at any point in the conversation, we coded it as a dichotomous indicator of *dominating/shaping the conversation*. We created a third, dichotomous variable to indicate whether the office at any point questioned the auditor's insurance status and this represents *misattribution*. We also created a dichotomous variable, named *Medicaid misattribution* to note whether they asked explicitly about Medicaid or CHIP.

### 2.4 Analytic approach

At the end of data collection, the team completed what has come to be known as initialization and construction processes.^35^ The team members read and reviewed all of the transcripts as one comprehensive document without any descriptors or identifiers and wrote reflective notes. Collectively, we first deductively reviewed the pre‐identified topics/themes, and then, we inductively added any themes that were missing from the list. We used this information to create a codebook that included descriptions of each theme. As part of the inductive process, Medicaid misattribution, reluctant acceptance, and implicit rejection were added as themes—all of the other, previously discussed measures were themes that we defined prior to data collection.

Coding of our qualitative data and the subsequent quantitative data entry was based on the resulting codebook and occurred at the blinded level. We hired a coder who was not familiar with the topic of the study and trained her to use the codebook to identify the categorical and binary variables that we analyze here. All coding was completed using Dedoose software, but the intercoder reliability function of Dedoose was not utilized because only one coder completed all of the conversion of qualitative data into our quantitative measures.

We analyzed the resulting quantitative data (combined with our administrative data) using StataSE 15, and significance was determined at an alpha level of 0.05. To explore the pilot study aims, we used logistic regressions on each of the binary outcome variables. We included all of the design variables in each of these models.

For our analyses related to the hypotheses, we performed a 1:1 matched pairs analysis using the mcc command for McNemar's test.^36^ These analyses focused on “discordant” pairs where auditors giving a Black cue received a different response than control auditors. The pairs where BOTH received a positive reaction or BOTH received a negative response are considered concordant pairs. The McNemar's tests focus on discordant pairs (ie, when a Black cue received a negative response and the control received a positive one). We present odds ratios and 95 percent confidence intervals based on these tests.

## 3 FINDINGS

### 3.1 Descriptive analyses

Table 1 provides descriptive information about offices’ behavior. Of the total 410 calls completed by auditors, 74 percent resulted in auditors being told without any qualifiers or restrictions that the office was accepting new patients (n = 302). At some point in the conversation, the person answering the phone asked most auditors about their insurance status (57 percent), but very few were specifically asked about Medicaid or CHIP (3 percent). About a quarter of the calls resulted in withholding behavior, and the office personnel discussed restrictions with auditors during just over one‐third of the calls.

**Table 1:** Frequency of explicit and implicit gatekeeping outcome variables (n = 410)

	Percentage	Number of calls
Acceptance	73.66	302
Withholding information	27.07	111
Dominating conversation	38.05	156
Misattribution	56.62	231
Medicaid misattribution	2.93	12

**Figure 2:**
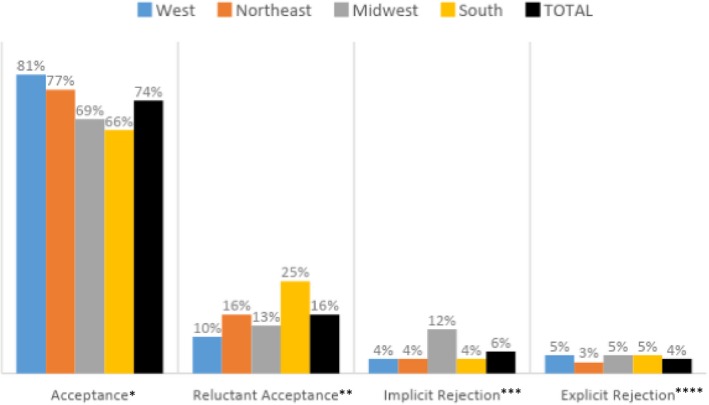
Regional variation in explicit gatekeeping and forms of rejections (n = 410) [Color figure can be viewed at http://www.wileyonlinelibrary.com/]

*Note:* *P = 0.05, *^*^ *P* = 0.02, *^**^ *P* = 0.02, *^***^ *P* = 0.84.

The most common response to calls across all regions is that the office is accepting new patients; however, the rate of acceptance varies across regions. As is evident in Figure 2, offices in the West have the highest rate of acceptance, regardless of race (81 percent). In comparison, the South and Midwest have much lower rates, respectively (66 percent and 69 percent). Perhaps more importantly, results show that the methods used to communicate that the office is not accepting new patients differ by region. For example, one‐fourth of the calls placed to offices in the South received a reluctant acceptance (a “yes, but…” response), more than in any other region. With a rate of 12 percent, offices located in the Midwest were twice as likely as offices in other regions to give an implicit rejection (the person answering the phone never said “yes” nor “no”).

### 3.2 Analysis of study design variables

Table 2 presents information from logistic regression analyses that were used to address two of our pilot aims. The results indicate that, overall, the study design variables explain little of the variation in any of the outcome variables (*R* ^2^ ranges from 0.02 to 0.04). Neither differing scripts nor order of the call was significantly associated with any of the outcome variables, except withholding information. In that case, the first script and first call were more likely to result in posing a question before providing an answer.

**Table 2:** Logistic regression exploring association between study design variables and outcome variables (standard errors in parentheses)

	Acceptance	Dominating conversation	Misattributions	Withholding information
Script 1	0.17 (0.29)	0.33 (0.26)	−0.02 (0.25)	−0.70 (0.29)*
Script 2	−0.24 (0.27)	0.44 (0.26)	−0.03 (0.25)	0.13 (0.26)
First Call	0.01 (0.23)	−0.00 (0.22)	−0.10 (0.21)	−0.53 (0.23)*
Low‐Segregation	0.02 (0.23)	−0.08 (0.21)	−0.43 (0.21)*	−0.07 (0.23)
Northeast	−0.25 (0.35)	0.82 (0.31)**	−0.62 (0.29)*	−0.16 (0.31)
Midwest	−0.67 (0.34)*	0.82 (0.32)*	−0.86 (0.29)**	−0.44 (0.33)
South	−0.89 (0.33)*	1.26 (0.32)***	0.12 (0.30)	−0.08 (0.31)
__constant	1.45	−1.34	1.43	0.199
Pseudo‐*R* ^2^	0.02	0.04	0.04	0.03

^*^ *P* < 0.05, ^**^ *P* < 0.01, ^***^ *P* < 0.001.

However, regional variation was evident in regressions on most of the outcome variables (with the exception of withholding information). In comparison with offices in the West, offices in the Northeast and Midwest were *less* likely to inquire about insurance but *more* likely to emphasize restrictions. Offices in the South were also more likely than those in the West to highlight restrictions. These variations compliment the regional differences noted in the descriptive analysis.

The last aim was to explore potential differences in the meaning of name cues vs linguistic cues. This exploration was completed as part of the testing of hypotheses and is discussed below.

### 3.3 Analysis of differences between “Black” auditors and controls

Table 3 presents the analyses performed to evaluate our hypotheses and to determine whether name cues and accent cues modify results. Our first hypothesis was that parents of patients perceived to be Black would be told that offices are accepting patients less often. Results of the McNemar's test show that a cue to Blackness trends in the correct direction, but is not statistically significant (odds ratio = 0.545; *P* < 0.08). When we distinguish between the type of cue used (name vs accent), results show that, compared to controls, auditors providing a Black sounding name were 64 percent less likely to be told that the office was accepting new patients (odds ratio = 0.36; *P* < 0.05).

**Table 3:** McNemar's test of differential responses from offices based on cues that the potential patient is Black

	Black Cue	Control	Odds ratio	Confidence interval
Acceptance	71%	76%	0.55	0.27‐1.10
Withholding information	31%	23%	1.79*	1.02‐3.14
Dominating conversation	40%	37%	1.38	0.72‐2.62
Misattribution	57%	57%	1.00	0.60‐1.66
Medicaid misattribution	4%	1%	7.00*	1.86‐56.89

	Black name	Control	Odds ratio	Confidence interval
Acceptance	75%	62%	0.36*	0.13‐0.99

	Black accent	Control	Odds ratio	Confidence interval
Withholding information	34%	20%	3.00*	1.41‐6.38

^*^ *P* < 0.05, omitted categories under “Black Name” and “Black Accent” are based on insufficient data or statistically insignificant results.

Our second hypothesis stated that auditors providing a Black cue would be more likely to be asked at least one question before the office answered their inquiry (withholding information). The results support this hypothesis. Overall, providing a cue that the caller is Black results in a 79 percent greater likelihood that they will be asked a question before the office personnel answers whether they are accepting new patients. Further analysis indicates that the Black accent cue primarily drives this result. An accent‐based cue is associated with a three times higher likelihood of being asked questions before receiving an answer.

Our third hypothesis focused on offices discussing restrictions as a way of dominating and shaping the conversation. The trend in the data goes in the expected direction; however, the results are not statistically significant.

Our fourth and final hypothesis predicted that parents of patients perceived to be Black would be asked questions about insurance coverage or Medicaid status more often (ie, subjected to misattributions). The results only partially support this hypothesis. There is no evidence that auditors giving a Black cue were more likely to be asked about insurance status. However, they were seven times more likely than the control auditors to be asked if their child was on Medicaid or CHIP (odds ratio = 7.00; *P* < 0.05).

## 4 DISCUSSION

Overall, our findings indicate that there is reason to further investigate discriminatory and gatekeeping behavior directed toward Black pediatric patients over the phone. Office personnel who spoke with our auditors’ delivered different messages to potential patients based on both Black sounding names and their parents’ Black‐accented English. Furthermore, the shape and form of these messages varied by region, and this variation should be taken into consideration in future studies of discriminatory behavior.

Specifically, our data indicate that the impression that a potential pediatric patient is Black may lead office personnel to (a) suggest that they are not accepting new patients, (b) delay answering the question until they find out more about the patient, and/or (c) question whether the patient is receiving subsidized coverage. These findings are consistent with existing telephone audit evidence of differential access to care based on subsidized insurance coverage status,^37^ socioeconomic status,^38^ and race.^24^, ^25^ However, all of these studies were conducted in individual cities, precluding an investigation of regional differences.

Our pilot study suggests that the ways an office verbally indicates that it is not accepting new patients vary by region. A small percentage (3 percent‐5 percent) of offices in every region explicitly state that, no they are not taking new patients. However, 1 in 10 calls to offices in the Midwest never received an answer to the question. We interpret this as an implicit or passive rejection. One in four calls to offices in the South were told “yes” but were immediately provided information indicating that the family might not be welcome in the office. These responses included statements such as “Yes, but where do you live? There may be another office closer to you,” or “Yes, but only if you can fax us his records from the previous physician.” Scholars should consider these regional variations in future studies.

Our results also indicate that both accent cues and name cues should be taken into account in future studies, as they may be associated with different types of discriminatory behavior that limits access to health care services. The different experience based on these cues is important on several levels. It highlights the heterogeneity of experience within the Black population. Recall that all of the auditors were Black women, so perceiving some as White shows that this type of discrimination can be fallible. It also indicates that, linguistically, there may be a certain level of “acceptable Blackness” similar to colorism^39^: just as Black people with darker skin tones often face more discrimination, those with a Black sounding name or speaking Black‐accented English may be beyond the acceptable linguistic level of Blackness.

The results also have more practical implications. In our data, a Black name was associated with fewer responses from the office that they were accepting new patients. In contrast, evidence of withholding information was driven by the Black‐Accented English cue. Previous research finds that Black‐accented English tends to be interpreted as a cue not only to race but also to social class.^40^ This indication of class may help to explain why accent could lead to more clarifying questions before they decide to accept the new patient. Our cue‐specific results indicate that explicit gatekeeping may be directed toward Blacks regardless of class (based solely on name) whereas implicit gatekeeping may be restricted to certain Blacks, in this case, those who are simultaneously perceived to be Black and working or lower‐class.

Finally, the most substantial differential treatment that we observed was related to misattributions that the Black caller had public health insurance. Black callers attempting to schedule a well‐child visit for their son were seven times more likely than White callers to be asked something like, “Is he covered by CHIP?” or “Do you have Medicaid?”. This form of gatekeeping could be consequential given that, in 2014, 60.4 percent of Black children were covered by public insurance while only 25.1 percent of White children were covered by public insurance.^6^ All of our auditors responded “no,” but what message would the office personnel give if the auditor had said “yes”? Future studies should vary auditors’ insurance status to empirically investigate this question.

This represents a limitation of our study, but there are several others, mainly because this was a pilot study. All of our auditors were Black women, which highlights the heterogeneity within the Black population but also makes our analysis conservative. Furthermore, our study cannot determine how real mothers would respond to the differential messaging that we observed, nor what is driving differential treatment by staff. Some would argue that the results indicate that front‐office personnel should be more diverse (to increase racial concordance)^41^ or should undergo cultural humility training.^42^ However, the gatekeeping behavior among staff may not be driven by their individual racial biases. It is equally likely that staff are serving in the role of street‐level bureaucrats,^42^ where they exercise discretion in applying office policy because their position in the office—and opportunity for advancement—requires them to identify patients who maximize the idea of success for the medical office. Future studies will need to engage provider office staff to explore the motivations behind the differential messaging that was documented in the current research and other recent studies.

Additionally, our study suffered from small sample sizes that limited our power to detect region‐specific or cue‐specific differences in treatment. The small sample size also limited our ability to investigate the influence of the type of practice or other characteristics of the practice such as size or time in operation. These factors should be considered in the design of more extensive, future national studies.

Some might argue that further research on telephone‐based discrimination would be outdated, as office‐patient communication is quickly evolving to primarily occur online. We have witnessed substantial increases in patient preferences for and use of automated appointment reminders, notifications via SMS and email methods, and online portals.^43^, ^44^ However, all of this online communication—even the initial patient registration—only occurs after patients have been assigned to practices. Furthermore, ethnic/racial minorities and those with lower levels of education are less likely to use all of these online means of communication,^45^, ^46^, ^47^ maintaining the relevance of telephone communication to racial health equity. When racial/ethnic populations do interact with offices online or via SMS, name‐based cues to race and ethnicity (if not accent‐based cues) would continue to be pertinent.

Overall, future studies are immensely important because the dearth of field studies on this topic represents a limitation in our existing knowledge base. “Experimental approaches to measuring discrimination excel in exactly those areas in which statistical analyses flounder. Experiments allow researchers to measure causal effects more directly by presenting carefully constructed and controlled comparisons.”^48^ Information from audit studies was instrumental to drafting and implementing housing policy to address racial inequity in access to affordable quality housing.^49^ Audit studies have the potential to generate equally actionable information on children's access to preventive health care services in general, and well‐child visits in particular. And the implications of this type of research could reach well beyond pediatric care. It is well documented that minority patients’ perceptions of negativity and discrimination affect their health behaviors. Patients’ reactions to discriminatory messages—based on both race and class—ultimately influence not only access to care, but also psychosocial communication, adherence to medical advice, and patient‐initiated early termination of treatment.^50^, ^51^ For all of these reasons, further research on the topic is essential.

## CONFLICT OF INTEREST

No disclosures or conflicts of interest.
